# Using the natural capital framework to integrate biodiversity into sustainable, efficient and equitable environmental-economic decision-making

**DOI:** 10.1098/rstb.2023.0215

**Published:** 2025-01-09

**Authors:** Amy R. Binner, Ethan T. Addicott, Ben Balmford, Brett H. Day, Mattia C. Mancini, Danny Williamson, Ian J. Bateman

**Affiliations:** ^1^ Land Environment Economics and Policy Institute (LEEP), University of Exeter, Rennes Drive, Exeter EX4 4PU, UK

**Keywords:** natural capital, biodiversity, economics, conservation policy, land use

## Abstract

One of Georgina Mace’s many transformational research contributions was to provide a universally applicable framework for incorporating any or all elements and connections of the natural environment within conventional economic decision-making. We apply this natural capital framework to consider the overall effects of a suite of land-use policy options intended to promote the conservation and renewal of biodiversity. Options considered include sharing, sparing, three-compartment sparing, rewilding and organic farming regimes. Each is assessed in terms of its impact on both domestic and global biodiversity. Reinforced by an empirical application considering land use in Great Britain, we show that while policy has prioritized sharing options, evidence supports land sparing and three-compartment approaches as more efficient, sustainable and equitable alternatives.

This article is part of the discussion meeting issue ‘Bending the curve towards nature recovery: building on Georgina Mace's legacy for a biodiverse future’

## 1. Extending the natural capital framework for biodiversity policy decision-making

The interdisciplinary contributions of Georgina Mace to the ecological and wider sciences are simply enormous and defy ready summary. Her ability to reach across disciplines and beyond academia into the policy sphere was similarly remarkable. The diamond-clarity characterizing her expertise in translating complex concepts to research collaborators across different fields, or to policy and decision makers, was unique. This paper takes as its starting point one of those interdisciplinary contributions, her work on the natural capital approach to decision-making, and extends those principles to a major focus of Georgina’s research: biodiversity.

**Figure 1.**
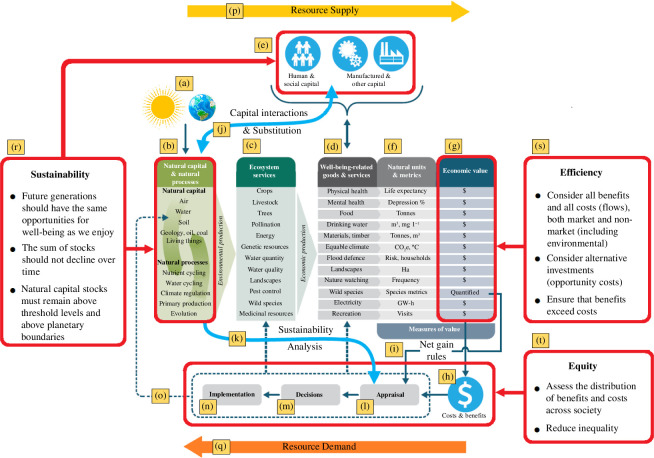
The natural capital framework for decision-making. Adapted from Bateman and Mace [1]. Sources: Sun png Timplaru Emil at https://www.vecteezy.com/free-png/sunshine; Earth png Muhammad Sana Ullah at https://www.vecteezy.com/free-png/globe-free.

Georgina’s work (with one of the coauthors of this paper) on the natural capital approach [1] was clear and generalizable, providing a universally applicable framework that incorporates any or all elements of the natural environment within conventional economic decision-making. Figure 1 provides an illustration of this natural capital framework. Here the ultimate source of resources, (a) the earth and energy inputs from the sun, drive the creation of (b) natural capital stocks, so called because they are: stocks, which can be drawn upon; capital, in the sense that they provide flows of potential services (and in some cases the direct provision of goods) of use to people and; natural, originating without human inputs. These stocks can be subdivided. Non-renewable resources (such as oil and gas) contribute to human well-being through their consumption, although the services they provide (e.g. energy) can be made sustainable by reinvesting sufficient proceeds of their use within alternative, reproducible, sources of those services (e.g. renewable energy) [2]. Alternatively, many natural capital resources are self-renewable (e.g. forests, fish, the atmosphere) provided they are not over-exploited. Together these various natural capital stocks enable the operation of a variety of natural processes crucial to life on earth (e.g. biogeochemical cycles). Note, however that connections between stocks mean that overuse of one (e.g. oils and gas reserves) can lead to impacts on another (e.g. climate change where atmospheric stocks are degraded, resulting in impairment of climate services).

Natural capital provides a wide array of (c) ecosystem services. While these are valuable inputs to production of (d) welfare-bearing goods and services, most production also requires inputs from other forms of capital (e). For example, human capital provides labour inputs while built capital can provide mechanical work. A practical problem, growing rapidly along with human population and economic development, arises from the limited availability of both ‘source’ (e.g. fish stocks) and ‘sink’ (e.g. the ability of the atmosphere to absorb carbon dioxide) resources (p), relative to the demand for welfare-bearing goods and services (q). These resource constraints define the essential economic problem of scarcity. Resource constraints mean we cannot enjoy unlimited quantities of all the goods that contribute to human well-being; trade-offs and decision-making become unavoidable.

A practical problem for decision-making is that goods and services are most naturally measured in an array of disparate units (f) that are neither commensurate with each other (how do we trade off tonnes of food with tonnes of CO_2_ emissions?) nor immediately relatable to well-being. In an attempt to address both aspects of this problem, economic analyses seek to convert these physical measures (f) to well-being as measured by economic value (g). The rationale here is that if we can estimate the value of—say—changing food production and the value of reducing carbon emissions in the same money metric, then both can be brought fairly into decision-making. Money represents the 'least-worst' of a range of imperfect measures [1], being—albeit incompletely—related to welfare (both as a present flow (money) and stock (wealth) of future well-being) and familiar to individual and policy decision makers (e.g. allowing the value of environmental improvements to be assessed in common units against the net benefits of other investments). Accordingly, a flexible set of economic methods have been developed for valuing many welfare-bearing goods, including those provided via markets and many non-market goods [1].

Once converted to these comparable monetary units, any changes to monetized goods and services (e.g. because of new policies) can be aggregated into a measure of net benefits to society (h). However, while economic methods can now robustly value the majority of goods and services, some are not amenable to monetization. In particular, despite recent advances [3], there are gaps in our current scientific understanding of the contribution of living species to maintaining ecosystem functions and the ‘non-use’ (e.g. existence) values of wild species. Therefore, the economic valuation of biodiversity benefits (e.g. the existence of species and the ecosystem functions they support) is not, in our opinion, sufficiently reliable for decision-making purposes. In the past, non-monetized goods have often been effectively excluded from decisions. To avoid this and ensure that biodiversity is not omitted from decision-making, we recommend rules (based upon both scientific evidence of ongoing loss and legislation seeking to reverse that trend) requiring that the effects of a policy or investment upon biodiversity should be non-negative. This obviates the need for valuation [1,4]. While imposing a rigid ‘no-loss’ rule upon the location of every investment or spending project could stifle change and is unlikely to be generally acceptable, an effectively imposed biodiversity ‘net gain’ requirement (i) has the potential to turn development into an engine for nature restoration. The costs associated with imposing a biodiversity net gain rule commensurate with attaining an ecologically determined nature restoration target provide a simple way to incorporate nature in economic cost–benefit analyses. However, care has to be taken to remember that these are the costs of ensuring the sustainability of biodiversity and may be quite different from its value.

While decision makers should have awareness of all stages of the framework, it is the Appraisal process (l) that is the major focus of decision-making. Appraisal of a policy option should be made with respect to three decision-making principles: Sustainability (r); Efficiency (s); and Equity (t). The domains that fall under each of these principles are highlighted in corresponding red boxes in figure 1.

The natural capital framework is ideally suited to the challenge of articulating the concept of sustainability. One approach (often referred to as ‘weak sustainability’) is to require that the overall economic value of all capital stocks should not decline over time [5]. This requires comparison of and effective substitutability between different capital types. Here, for example, natural capital stocks can be depleted provided that, say, human and manufactured capital stocks are appropriately built up (indeed, this is what has happened for much of human history) via (j) the interactions and substitution between capital types. However, depleting natural capital stocks below ecological thresholds (sometimes referred to as planetary boundaries [6]) risks the potential for irreversible ecosystem collapse, incurring devastating welfare losses owing to non-substitutability [7]. True sustainability (often referred to as ‘strong sustainability’) therefore requires *both* that the overall economic value of all capital stocks should not decline over time *and* that resources are maintained within a ‘safe operating space for humanity’. In the case of natural capital, this latter constraint requires that the physical ecological thresholds of natural assets be respected and that there is no net loss beyond those planetary boundaries. This concern is heightened by recent research showing that many stocks have already been pushed beyond safe operating levels [8] and that continued over-use of natural resources may push natural capital stocks beyond tipping-points, triggering sudden, non-linear accelerated losses [9]. Given this, (k) sustainability analysis, to ensure that ecosystem processes are not compromised, is an essential part of (l) the appraisal of policy and investment decisions.

Efficiency (s) requires that policy options should be appraised across all the benefits and costs that a policy generates. This includes effects arising outside the immediate geographical focus of the policy; if those changes induce impacts elsewhere, they should be counted. Where possible, all these effects should be robustly valued, irrespective of whether the goods concerned are traded in markets or not. Where robust valuation is not possible (as in the case of biodiversity) then net gain requirements should be imposed in these cases. A further obvious (but often omitted) requirement is that appraisal should include a range of possible alternative decisions or investments. Consideration of all benefits, costs and alternatives should extend until the costs of further appraisal outweigh the likely net benefits arising from consideration of further impacts and options.

Almost every policy has winners and losers, and hence equity (t) implications. Arguably, distributional issues might best be handled separately via transfers between members of society based on the assumption that if decisions are efficient then equity is best addressed through economic tools such as redistributional taxes (e.g. de Scitovsky [10]). However, there may be practical arguments for making a policy overtly equitable if a failure to do so threatens the viability of that policy (e.g. an energy tax that hits the poor relatively harder than the rich might be made more acceptable if revenues are recycled in a redistributive manner, say by funding free insulation for disadvantaged households). Further, if justice and fairness are considered as criteria for assessing sustainability, separating equity from efficiency also cleaves notions of sustainability from socio-economic development. Intragenerational efficiency and equity are necessary, but not sufficient criteria for sustainability and consideration of intergenerational effects (‘the ability of future generations to meet their needs’ [11]) are vital elements of the natural capital approach to decision-making.

This simultaneous consideration of the sustainability (r), efficiency (s) and equity (t) implications of a policy marks out the challenge of appraisal (l). This step must also consider how alternative modes of implementation (n) would affect the consequences (o) of the decision and how those consequences in turn ripple dynamically through to further changes in stocks, services, goods and their values (a process of iterative appraisal referred to as ‘double-loop learning’) [12]. The natural capital framework provides the necessary information required to meet such challenges in an objective, consistent and transferable manner.

Comprehensive appraisal (l) should include the impacts of alternative modes of policy implementation (n) to ensure informed decision-making (m). This in turn passes through the chosen optimum approach to implementation (n) to yield outcomes (o) that support Sustainability, Efficiency and Equity: the SEE Principles underpinning the natural capital framework.

## 2. Assessing conservation policy through a natural capital framework

The consideration of all capital stocks, service flows, goods and values captured within the natural capital framework makes it highly adaptable and applicable to any decision situation. The framework permits sustainability analyses (k), efficiency assessments (s) and equity concerns (t). Furthermore, the wealth of information that application of the framework encourages allows policy makers to assess decision options from two distinct perspectives: that of social well-being, where all elements of the framework are relevant; and from the viewpoint of the private decision maker, where only a subset of those elements are considered, most pertinently the private benefits and costs relevant to such actors. The latter, more restricted perspective also reveals important insights to the policy maker, showing the gains and losses of a potential change as viewed by individuals and private businesses and indicating their likely response to such changes. Through comparison of these private responses with corresponding social well-being impacts, the policy maker is able to refine policies to attain the optimal feasible outcome within a political system where the majority of resources are privately owned and changes therein can only be incentivized rather than dictated.

These characteristics of the natural capital framework make it especially suitable for the incorporation of biodiversity within decision-making. The emphasis upon sustainability is particularly important given ongoing biodiversity loss. Similarly, the fact that change in biodiversity is very rarely and imperfectly reflected in market-priced goods makes the efficiency dimension of the natural capital framework, with its requirement to include non-market benefits and costs, a vital requirement for bringing impacts on wild species and their role in delivering ecosystem services into consideration. Likewise, the natural capital framework explicitly recognizes that the more general value that wild species provide cannot (currently) be monetized, and instead it includes net gain requirements for biodiversity. Finally, given that the costs and benefits of conservation are far from equally distributed, the framework’s emphasis on equity is again important.

Within the terrestrial environment, the main driver of the habitat loss and degradation underpinning the global biodiversity crisis is agriculture [13]. In response to this, a variety of ‘agri-environmental’ policies have sought to incentivize changes in farming to promote conservation. However, in seeking to improve biodiversity conservation, these policies actually impact a wide range of natural, produced and human capital stocks, driving changes—positive and negative—in both biodiversity and a wide range of market and non-market, environmental and manufactured goods and services. The natural capital framework emphasizes the need to consider all of these effects, both the direct focus of a change and its indirect consequences, when appraising policy options. In the remainder of this section, we overview the diversity of agri-environmental conservation policies in use, summarizing their various impacts not just upon biodiversity but also on food production and the knock-on, indirect consequences that they generate. In §3, we apply stylized versions of this framework to appraise conservation policy in Great Britain by modelling anticipated changes to domestic food production, opportunity costs, total conservation area and species richness.

**Figure 2.**
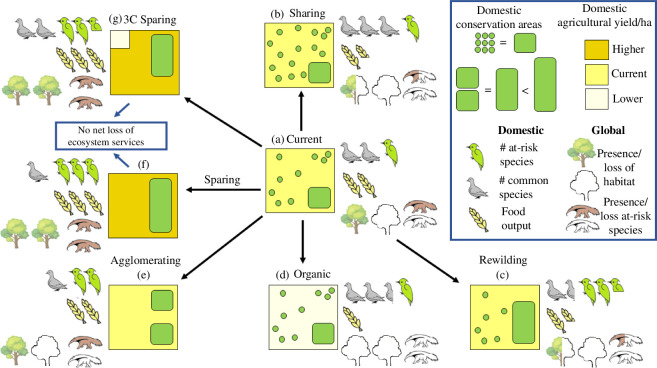
The spectrum of agricultural and conservation land-use types, which are not all mutually exclusive and may be pursued in combination. The indicated outputs of common and at-risk domestic/global species, food outputs and global habitat loss are based on our reading of the literature cited/discussions with relevant experts and are only intended to provide relative comparisons between land-use types. 3C Sparing, ‘Three-Compartment Sparing’ (see end of §2). Tree image by https://www.rawpixel.com/ on Freepik; other bird, food and giant anteater images by Sian Brooks.

Figure 2 illustrates the spectrum of agri-environmental conservation policies, some of which may be pursued in combination, and corresponding land-use types typical of many developed countries. The centre of figure 2 shows the (a) current rural land use typical of many developed countries. Here landscapes are typically dominated by agricultural production, with a relatively small number of larger, often vestigial, contiguous natural areas (shown by the green rectangle). Within agricultural areas, taxpayer-funded policies support a number of small-scale habitat features (shown as green dots) including the margins and corners of fields. These often benefit species that are already relatively common [14,15], and in general making areas of agricultural land more ‘wildlife friendly’ tends to be of benefit to species with large global ranges [16]. However, those that require larger contiguous areas, such as woodlands, marshlands and heaths, find such farmed environments challenging, even with these fragmented small-scale habitats [17]. Furthermore, in many such developed countries (with the UK being a prime example, which we focus on subsequently), the average productivity (yields) and total food output of such systems fail to satisfy burgeoning demand, resulting in substantial agricultural imports. These imports are typically sourced from lower-cost, more climate-sensitive and less-developed countries where food production imposes additional threats to species (i.e. a larger biodiversity footprint) [18–20]. Source countries may not necessarily be richer in resources or biodiversity; however, changes in net imports can impact biodiversity loss far afield owing to the globalized nature of the food supply chain [21]. In effect, shortfalls in domestic production drive global biodiversity loss via demand for imports.

For decades now, policymakers in the UK, Europe and increasingly globally have sought to address conservation goals by allocating the majority of their agri-environment budgets towards subsidizing farmers to make areas of agriculture more ‘nature friendly’ (e.g. by the creation of more of these small-scale habitat features within an overwhelmingly agricultural landscape). The resulting approach, which approximates ‘Land Sharing’ (b), typically promotes the abundance of common and rarer species, which can adapt to such areas. Common species may of course be those that underpin a greater share of ecosystem service delivery [22] and the natural capital framework makes clear that these benefits (some of which, such as recreation, can be robustly monetized [23]) should be accounted for.

The attraction of Sharing is understandable when analysis is restricted to the area in which such policies are applied; when analysed at, say, a farm or local environment level, the introduction of Sharing increases local biodiversity. However, such ‘focusing’ errors [19] fail the basic efficiency principles of the natural capital approach, which require that all the impacts of a policy should be considered, including knock-on effects arising elsewhere. First, and as acknowledged by the UK Office for Environmental Protection [24] and EU Auditors [25], European and UK agri-environment policies in the round, which broadly conform to Sharing-like policies, have failed to halt declines in overall domestic biodiversity within those countries applying such measures. Indeed even farmland species, which policies often aim to conserve, have continued to decline [26,27], although significant domestic improvements in populations of some bird and butterfly species have been evidenced under Sharing-style policies in parts of the UK [28–33]. Second, these ongoing domestic biodiversity declines become more concerning when the global effects of Sharing are considered. While Sparing outperforms Sharing, regardless of the level of total agricultural production required, gains from Sparing are particularly large when more production is required [16,34]. Therefore pursuing a strategy of lower yields at a time when global food demand is at an all-time high and growing [35] seems likely to backfire. The additional imports required to compensate for Sharing-induced reductions in food production lead to greater negative impacts on biodiversity and natural habitats elsewhere [18], including in high biodiversity regions [13]. Europe now appropriates 1 ha of cropland abroad for each 4 ha of domestic cropland [35], while the EU’s crop imports from 1990 to 2014 have generated over 11 million ha of habitat destruction in some of the world’s most biodiverse ecosystems [19,36]. As a conservation policy, Sharing may have generated modest local gains, while likely fuelling biodiversity loss elsewhere.

The efficiency principle of the natural capital framework highlights government unwillingness to consider either the domestic effectiveness or the global biodiversity impacts of conservation policies. This latter impact is of particular concern given the recent Convention on Biological Diversity 30 × 30 Declaration, signed by nearly every country in the world [37]. Its commitment to set aside 30% of the world for nature by 2030 (Targets 2 and 3 of the Declaration) is an important step forward in recognizing the seriousness of the biodiversity crisis, but in implementing this policy governments need to consider the impact that this may have on domestic food security and the likelihood of global spillover impacts on biodiversity.

Another, and recently popular, response to the biodiversity crisis has been (c) Rewilding schemes. Here, rather than opting for small-scale, scattered habitats, land is typically taken out of farming in larger contiguous areas and restored to a semi-natural state. Rewilding has often been met by objections regarding the societal acceptability of restoring large swathes of habitat and the (re-)introduction of species on a variety of grounds [38]. Nonetheless, Rewilding can benefit those most at-risk species that require large areas of contiguous natural habitats and the restoration of ecosystem functions and disturbance [39]. However, Rewilding alone still reduces total agricultural production, potentially increasing demand for imports and again damaging biodiversity elsewhere. Taken on its own, Rewilding cannot be a panacea for the biodiversity crisis.

Conversion to (d) Organic farming has a longer pedigree but looks likely to perform more poorly than either Sharing or Rewilding in terms of its overall impact on biodiversity. In general, terrestrial species in those areas converted from intensive to organic farming can benefit from the removal of manufactured fertilizers and pesticides, with some increases in bird abundance and species richness observed [40]. However, impacts upon aquatic species are less obviously enhanced as phosphorus loadings of livestock farms remain elevated, whether downstream of conventional or organic farms [41]. More significantly, the substantially lower yields synonymous with organic farming raise major risks of global leakage of biodiversity losses. Switching to organic farming across all of England and Wales is estimated to cut agricultural calorific food-energy output by 40% [42]. While the forced introduction of organic farming has proved highly contentious in the developing world [43], its popularity is growing in richer countries, with the EU and Japan committed to a target of 25% of food production from organic farming [36]. Without further changes, the major reductions in domestic food production that this is expected to cause (with estimates exceeding 30% for France [41]) is likely to have marked impacts on land use and biodiversity elsewhere. Those choosing organic foods for their perceived environmental credentials might be surprised at the wider negative consequences for wildlife of their choices.

Given the potential downsides of Sharing, Rewilding and Organic options, what other alternatives are there? One highly cost-effective option would be to reduce subsidies for fragmented small habitats and instead focus public funding towards the creation of large (e) Agglomerated blocks of contiguous habitat. Large areas of habitat have lower perimeter-to-area ratios and as such are less impacted by edge effects and may therefore hold larger populations of those species that require more natural habitats [44]. It is worth caveating that while the general trend is for species to do better in larger areas of continuous habitat, some species appear to prefer a greater length of habitat edge [45]. Nevertheless, the variety of environments that arise in larger habitat blocks could support a greater diversity of specialist species [46]. This is why a large forest has more wildlife than the same area of small separate woodlands. Hence, even if we hold the non-farmed fraction of the landscape constant, by lumping habitat patches together this Agglomeration effect will enhance the prospects of at-risk species reliant upon larger, consolidated habitats. Within England, the transition of farm subsidies from a focus on production to the Public Money for Public Goods basis envisaged under the 2020 Agriculture Act has the potential to transform agri-environmental schemes, moving away from poorly performing Sharing policies that have tended to fund the creation or maintenance of small, isolated fragments of habitat, and instead supporting the creation of larger, connected habitat blocks. Focusing these agglomerated habitats to those areas of greatest wildlife value, rather than distributing them across the country, would also create the network for nature that is essential to bend the curve on biodiversity loss [47,48]. Keeping constant the area of non-farmed land and associated publicly funded subsidies also raises the prospect of delivering greater biodiversity gains for the same cost, improving value for money to the taxpayer. However, care needs to be taken regarding the speed of any transition. Rapid wholesale change from current to agglomeration policies could cause challenges in the short term (which may be the most relevant timescale for policy makers) if this results in the immediate loss of smaller habitat patches while newly agglomerated habitat takes time to mature and be colonized. A phased approach to agglomeration is therefore likely to be preferable.

While larger, more consolidated areas of natural habitat offer conservation gains over the present-day situation, they may still imply a reduction in food output, with consequent increases in imports and overseas biodiversity loss. This can be driven by agglomeration alone if, in agglomerating, higher productivity areas are taken out of production. This concern is directly addressed within the (f) Sparing approach to conservation, which argues that a move towards higher-yielding agriculture on a smaller land footprint offers the prospect of bending the curve on biodiversity loss not just locally, but globally [17,49]. The logic of the Sparing approach recognizes that any policy that reduces food production in one area will increase it in another. Sparing strategies address this by promoting higher-yielding agriculture as the cornerstone of conservation [50], boosting domestic food production to allow farming to be focused onto smaller areas, which spares land for wildlife while avoiding the need to increase imports with their associated global biodiversity losses [17,49]. To achieve this potential, Sparing requires changes in policy to promote increases in yield but expressly link these to the provision of conserved habitat [51]. Furthermore, the forms of yield increase must themselves be sustainable, avoiding strategies that deplete soil fertility or increase environmental impacts; we cannot hope to conserve species by destroying the environment.

As emphasized in Target 10 of COP15 of the Convention on Biological Diversity agreement [37], any increase in agricultural yields needs to be delivered without exacerbating the environmental impact of farming. A number of promising routes for such sustainable intensification have been developed, including improved animal husbandry [51,52]; accelerated livestock breeding and crop selection [53]; vertical farming [54]; traditional co-cropping systems [55]; sophisticated deployment of native plants capable of drawing pest species away from crops [46]; terrestrial closed-system and naturally low-impact aquaculture [56]; microbial fermentation [57] and microbial protein production [58]. Economic and property rights instruments can help catalyse such yield gains [59]. The effectiveness of these technologies in improving yields and therefore safeguarding areas for nature is not generally dependent on dietary change. Indeed, reductions in total demand bought about by Global North citizens moving to less-damaging diets would lower total production targets, but Sparing would still outperform Sharing [16,34]. That said, it is of course the case that reductions in demand bought about through reduced demand for meat and dairy would enhance the biodiversity gain of any strategy [35,60], and changes in the agricultural system cannot therefore be seen as a substitute for changes elsewhere in the food system. The target here is to raise yields at limited cost to more common and widespread species so that more local land can be taken out of agriculture and into larger blocks of natural habitat [49,61] (f), and/or domestic food production is increased, reducing food imports and hence pressure on species and habitats elsewhere in the world.

Of course, yield increases do not inevitably lead to more land for conservation. Indeed, critics of Sparing highlight the potential for productivity gains to lead to increased resource use in the local area (the Jevons paradox [62]) dragging more land into production. While there have been several relevant studies [17], evidence of such extreme rebound effects is mixed, with a number of analyses omitting key drivers of agricultural expansion such as population growth, the balance of food imports and socioeconomic time trends [63]. More commonly, efficiency increases are partially rather than wholly offset by rebound effects such that reductions in farmed area are less than proportional to yield increases [64]. Nevertheless, yield increases do generate net release of land and this could be enhanced through the introduction of measures linking subsidies for yield improvements to the conservation or restoration of natural habitats^1^ . The observation of so-called ‘passive sparing’ occurring without policy intervention [65] suggests that such subsidies might well be effective.

Moreover, increases in total domestic food production also reduce reliance upon imports and their associated biodiversity impacts [66]. Realizing the full potential for yield growth to spare land for conservation requires the development of explicit policy or economic instruments that couple support for agricultural innovation and adoption of high-yield technologies with requirements for large area habitat creation [51,67]. In the context of Great Britain, these policies could include changes in the ways that agri-environmental subsidies are allocated to encourage land sparing, or offering subsidized yield-increasing technologies (e.g. precision agriculture, indoor aquaculture) conditional on habitat conservation [51,68]. When viewed through the holistic lens of the natural capital framework, subsidizing low environmental impact increases in farm yield and food production may well be the most effective approach to delivering overall improvements in biodiversity conservation.

The benefits of achieving Sparing through yield increases appear to be substantial. An expanding number of studies from around the world—which typically develop population density–yield curves and then model different land-use options for a given landscape and production target—have shown that to meet any given level of overall food demand, Sparing can deliver greater biodiversity gains than Sharing, especially for less-resilient specialist species that tend to be of greater conservation concern [51,61,69]. Furthermore, recent results from examining survey choices by British farmers presented with a range of conservation policy and subsidy payment alternatives show that Sparing approaches deliver the same levels of biodiversity benefits at 79% of the food production cost and 48% of the taxpayer subsidy cost of Sharing [68]. Given that reductions in the cost of delivering conservation mean that greater gains can be delivered for any given budget, this suggests that Sparing may be both more effective and economically efficient than Sharing. In-depth field assessments from five continents quantifying how the abundances of over 1500 species of birds, insects and plants change with farming practices have shown that, for the large majority of species, implementation of Sparing policies would outperform Sharing [17]. Importantly, the divergence in outcomes is so great that this overall result holds even if additional policy mechanisms to limit rebound effects are not place [70].

These empirical findings provide compelling evidence that Sparing outperforms Sharing in terms of its biodiversity benefits. Furthermore, in comparison to Sharing, a Sparing approach can also deliver significantly greater ecosystem service co-benefits, including greenhouse gas (GHG) removal and storage services and recreation benefits [71,72]. Other potential advantages of Sparing include relatively greater domestic (and exporter) food security [68] and potential employment benefits associated with a move to lower environmental impact food production technologies and large-scale habitat restoration [73,74]. Such benefits directly address popular concerns regarding the removal of land from productive agriculture on the grounds that it weakens food security and rural employment [75].

More recently, a further approach has emerged: (g) ‘Three-Compartment Sparing’ (3C Sparing in figure 2) [76]. Unlike ‘pure’ Sparing, Three-Compartment Sparing allocates some of the spared land, facilitated by high yields elsewhere, to very low-yield farming. In so doing, so-called ‘farmland specialist’ species that are dependent on disturbance (and since the widespread extinction of many megafauna relies on human-induced habitat perturbations [17]) may benefit as compared with a pure Sparing strategy. Indeed, recent research has shown that this blended approach may outperform not only Sharing, but also pure Sparing [76,77].

## 3. Applying the natural capital framework to comparison of conservation policies: a case study of Great Britain

Socio-ecological systems are complex and failure to include that complexity within policy and decision-making is a major cause of the environmental problems challenging humanity. The natural capital framework provides a roadmap toward incorporating this complexity within decision-making systems. While decisions should include all the multiple consequences of change, for clarity of exposition we demonstrate the utility of the framework by considering two important outcomes—food production and wild species—within the land-use system. Specifically, we explore the outcomes generated by alternative strategies for agri-environmental policies geared towards conservation through an illustrative application for Great Britain. Each strategy is compared against a no-policy-change common baseline of current land use with no change in either food yield or conservation. All analyses, baseline and alternative scenarios embrace spatial variation across the country and temporal variation to 2060, capturing climate change over that period (see subsequent details).

The no-policy-change baseline is separately compared with three alternative future strategies reflecting the Sharing, Sparing and Three-Compartment Sparing policies presented in the previous section. The Sharing strategy is simulated through policies that target the conversion of a quantity of land from agriculture to conservation through public subsidy. In any given 2 km cell, any portion up to all of the agricultural land in that cell could be selected for conversion to conservation, and in our analysis we examine the effects of varying the total area across all cells up to 600 kha nationally. This improves biodiversity domestically but also results in a reduction in agricultural output, which increases food imports. This in turn raises the prospect of telecoupling mediated by the global food system, resulting in overseas habitat reduction and leakage of biodiversity loss internationally.

As noted above, the initial focus for Sparing is on increases in food productivity allowing land to be taken out of agriculture for conservation without an overall reduction in food output, thereby avoiding the potential for leakage of biodiversity loss inherent in the Sharing approach. Our simulation of Sparing follows Phalan *et al*. [51], in which per hectare agricultural yields are increased through publicly funded subsidies of the necessary technology, in exchange for land being moved into conservation. The spatial sensitivity of our underlying model allows for variation in initial productivity across areas to which we apply a series of yield increases up to 5% nationally. As these are small in magnitude we assume that they do not impact in-field biodiversity; however, if land use remained constant then they would of course generate greater aggregate output. The Sparing strategy is then applied to each yield increase, removing a corresponding area of land into conservation such that the overall change in food output is zero. Ensuring that there is no change in total domestic food production means that there is no potential for international leakage of biodiversity loss via increased imports of food.

The final strategy that we consider is Three-Compartment Sparing. Here again, public funds are used to stimulate technological improvements to yield, raising productivity and permitting land to be spared into conservation without reliance upon higher imports. However, as shown in figure 2, not all the spared land is assigned to natural habitat. Following seminal work in this area [76], we assume that the area taken out of conventional agriculture under the Sparing scenario is now divided, with two thirds completely given over to nature conservation as before, while one-third (chosen to be that with the lowest output) is allocated to low-yield agriculture.^2^ For consistency we assume that the low-yield agricultural land will enjoy the same, albeit small, proportional increases in output over the time period of our analysis. Added to the productivity increases elsewhere on each affected farm, this results in a minor but positive increase in total food output and corresponding small reduction in imports.

To examine how each of our three strategies might perform across different conservation land uses, each is examined for conversion to (i) woodland, with a majority of broadleaf trees, or (ii) semi-natural grassland. This then yields six land-use scenarios. Furthermore, as the natural capital framework emphasizes, the way in which a policy is implemented can have a major impact upon the outcomes from that policy (see (n) in figure 1). Specifically, the extent to which spatial variation in the natural environment and across farms is incorporated into policy implementation can significantly alter the outcomes of policy change. To allow for this, each of the scenarios is implemented in three ways that vary the selection criteria used to determine which areas of land are chosen for conservation:

- *Equal Allocation:* Here a given level of land-use change is equally allocated across the entire case study area. This amounts to dividing the total area of additional conservation equally across Great Britain (in our analysis represented by 57 230 grid cells each of 2 km square). Under our Sharing scenario, equal areas of land are moved from farming to conservation in each cell, reducing agricultural output. Under the Sparing approach, as productivity increases so land is allocated from farming to conservation to the point where overall agricultural output is returned to its level prior to the productivity gain. This approach is also used for Three-Compartment Sparing, although the marginal increase in output on low-yield agricultural land results in a small increase in food production over time.
- *Cost Minimization:* Farms are ranked from lowest to highest agricultural output per hectare and the least productive (i.e. lowest subsidy cost) farms are selected for conservation schemes (an approach currently being considered by government for targeting net zero woodland planting). Under Sharing-style area-targeting, the lowest-cost farms are enrolled into the scheme until the target quantity of conserved land is met. Under Sparing, at each level of productivity increase, the lowest-cost farms are spared for conservation until the overall production of agricultural output is the same as prior to the yield increase. This approach is also used for Three-Compartment Sparing, although the marginal increase in output on low-yield agricultural land again results in a small increase in food production over time.
- *Biodiversity Maximization:* Farms are ranked from highest to lowest in terms of the gain in biodiversity that creating habitat in those locations would deliver. Those sites that deliver the greatest gains in species occupancy are then selected for conservation. Again, the total area that is taken into conservation differs. Under the Sharing approach, areas are selected for transition out of agriculture until the target quantity of conservation land is met. Under Sparing-style productivity-facilitated conservation, areas continue to be selected until agricultural output is reduced to be the same as prior to the increase in productivity. This approach is also used for Three-Compartment Sparing although the marginal increase in output on low-yield agricultural land again results in a small increase in food production over time.

In all cases, the land taken out of conventional farming is given over to conservation. For Sharing and pure Sparing, this means the creation of natural habitat (either grassland or broadleaf woodland). For Three-Compartment Sparing, two-thirds of the land taken out of farming still goes to natural habitat, while the remaining one-third goes into low-yield agriculture suited to farmland specialist species.

The impacts of land-use change upon biodiversity are captured through use of the UK Joint Nature Conservation Committee (JNCC) biodiversity modelling framework (following Croft *et al*. [78]), which links biophysical land characteristics, climate-related variables and resulting land use to measure change in species richness for 100 species defined as at-risk by the IUCN Red List.^3^ Specifically, the priority species model used in the analysis is based on a species distribution modelling framework developed by the JNCC [79], parameterized at the 2 km grid cell resolution. Using species presence data from the National Biodiversity Network Atlas [80], the diversity (measured using probability of occurrence) of each species is predicted as a function of climate, topography, land use and soil characteristics, using an ensemble of seven popular modelling strategies: bioclim, boosted regression trees, general linear model, generalized additive model, kernel support vector machine, Maxent and random forest. The modelling framework takes an iterative approach, with the best model selected according to the Receiver Operating Characteristic curve over 100 repetitions with different subsets of the data. The final model for each species is an average of the predictions of probability of occurrence from the 100 best models.

To predict the biodiversity impacts of different policies, spatial, temporal and climate-sensitive modelling of land use and land-use change are undertaken using the environmental–economic Natural Environment Valuation (NEV) modelling suite [4,81,82]. This brings together the drivers and physical consequences of land-use change captured within the natural capital framework (figure 1), including a high-resolution agricultural model synthesizing long time series along with spatially disaggregated data on the biogeophysical, agricultural output and economic characteristics of farms. The resulting predicted changes in land use are then used to model species’ probability of occurrence for each 2 km grid cell. These are then summed across all cells and species to deliver the ‘Gain in Occupancy’ measure we use to reflect a policy’s impact upon biodiversity.

The initial land-use baseline was taken from the Centre for Ecology and Hydrology (CEH) 25 m resolution raster [83]. Our biodiversity results are changes in the species richness in each grid cell averaged from 2020 to 2060 and accounting for cell-specific biophysical land characteristics, climate and climate change-related variables, and resultant land use under each policy strategy. These are compared with the food production for each cell, period and strategy. Cell-specific agricultural opportunity costs are also calculated as the net present value of food production flows between 2020 and 2060 using the UK's HM Treasury 3.5% discount rate. We account for the predicted impact of climate change on agricultural output and species distribution over this period using the UKCP18 6.5 climate projections for temperature and rainfall [84].

## 4. Results

**Figure 3.**
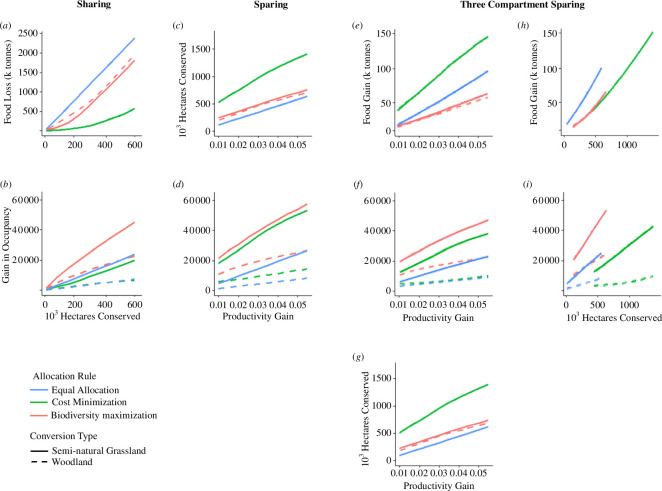
Conservation, biodiversity richness (change in occupancy), productivity and food output under three agri-environmental conservation policies, each implemented through three land allocation rules, and all applied to two conservation land-use types. Panels (*a*) and (*b*) show results from applying a Sharing approach where conservation area targets are set. Here (*a*) shows changes in domestic food output under different conservation area targets, while (*b*) illustrates corresponding species richness gains (the gain in species occupancy for 100 at-risk species summed across all grid cells). Panels (*c*) and (*d*) show comparable results from applying a Sparing approach where increases in productivity (varied from 1% to 5% increases in yield) allow land to be moved into conservation up to the point where total food output is kept constant. Here (*c*) shows the potential for land sparing into conservation under constant total agricultural output under varying productivity gains, while (*d*) illustrates corresponding species richness gains as productivity varies. Panels (*e*), (*f*) and (*g*) show results from a Three-Compartment Sparing approach. Here again productivity is increased, allowing land to be spared from conventional agriculture without lowering food output. Now one-third of that land is converted into low-yield agriculture suited to farmland specialist species, while the remaining two-thirds are consigned to natural habitat. Implications of the results from panels (*e–g*) are considered in panels (*h*) and (*i*) which show the relationships between area conserved and (*h*) corresponding change in food output and (*i*) biodiversity. All three strategies are implemented under three rules for allocating the location of conservation land: equal allocation (shown in blue); cost minimization (green); biodiversity maximization (red). Furthermore, each strategy and allocation is assessed for two forms of conservation land-use; conversion into semi-natural grassland (solid line curves) and woodland (broken lines).

It comes as no surprise that setting targets for land conservation generates biodiversity gains (as measured by the diversity of 100 at-risk species based on their modelled probability of occurrence) and has the potential to generate agricultural production losses if these are not addressed. Figure 3 reveals the nuances associated with the policy scenarios described above.

Unlike previous analyses of Sharing and the two variants of Sparing, we do not maintain aggregate domestic production across the three strategies, such that comparisons of domestic biodiversity must also consider international leakage through changes in domestic food production. Sharing policies remove land from production with no compensating increase in yield, resulting in lower domestic food production and hence higher imports, which in turn raise the potential of biodiversity loss leakage internationally. Conversely the increase in yields under Sparing maintain total domestic food production, ensuring that imports remain constant and biodiversity loss leakage is avoided. Our productivity assumptions imply a small net increase in total production under Three-Compartment Sparing (an order of magnitude smaller than the losses associated with Sharing; compare panels (*h*) and (*a*) in figure 3), again avoiding an increase in imports or biodiversity leakage.

While results for all policy types are reported for each of the three implementation rules, in practice the *Equal Allocation* approach is most commonly associated with the area-targets used in Sharing policies and it is this permutation that deserves particular attention here. By contrast, productivity-facilitated Sparing conservation policies are typically implemented via a *Cost Minimization* approach and again this is most relevant to such policies.

Panels (*a*) and (*b*) in figure 3 report results for the Sharing analyses. Here (*a*) quantifies the food output losses that need to be offset (e.g. via imports) as the area of Sharing-style conservation increases. Contrasting this with (*b*) reveals that Sharing via *Biodiversity Maximization* (prioritizing conservation in those areas which deliver the greatest gain in occupancy; red lines) delivers significantly greater increases in biodiversity than does the *Equal Allocation* approach (blue line) typically adopted for Sharing policies. Contrasting conservation land uses, semi-natural grasslands (solid lines) deliver substantially greater biodiversity benefits than woodlands (broken lines) across both of the latter allocation approaches. However, returning to (*a*), all Sharing permutations incur reductions in total food output, increases in imports and hence biodiversity loss leakages internationally.

Panels (*c*) and (*d*) report results from the Sparing analyses. Here (*c*) quantifies the impact of productivity gains in terms of the area potentially spared into conservation while keeping total food production constant. At a 5% increase in yield, the *Cost Minimization* approach most suitable for Sparing results in more than twice the area conserved under Sharing applied via its typical *Equal Allocation* approach. However, it is the biodiversity implications that are of particular importance, and panel (*d*) translates Sparing yield increases into biodiversity richness gains. These results show, independent of the habitat being created, that the *Biodiversity Maximization* implementation of Sparing outperforms *Cost Minimization,* while an *Equal Allocation* approach is inferior in all cases. Comparing biodiversity outcomes from the yield increases for Sparing (*d*) with the area increases for Sharing (*b*) shows that, for semi-natural grasslands implemented via *Biodiversity Maximization,* a yield increase of just under 4% delivers the same biodiversity gain as a 600 000 ha increase in conservation area under Sharing. These gains are roughly halved under *Equal Allocation* implementation, irrespective of whether Sparing or Sharing approaches are used (although the former does avoid international leakage of biodiversity losses), a result that underlines the folly of ignoring environmental and economic variation in this commonly applied policy mechanism. However, the contrast between Sparing and Sharing is most marked under the *Cost Minimization* approach, where a yield increase of just over 1% under Sparing outperforms even a 600 000 ha area increase under Sharing. All of these comparisons are solely in terms of domestic biodiversity gains and it should be remembered that the Sparing approach avoids the international biodiversity loss leakage problems of Sharing (as per figure 2). At higher yields, Sparing under *Cost Minimization* performs almost as well as the same policy implemented via *Biodiversity Maximization*, a result that is likely to be of interest when environmental policy budgets are limited. Again semi-natural grassland endpoints outperform woodland in terms of domestic biodiversity gains, an issue we return to below.

Three-Compartment Sparing delivers the same land conserved as Sparing (i.e. panels (*c*) and (*g*) are identical) and marginally increases domestic productivity (panels (*e*) and (*h*)) such that biodiversity loss leakage is again not an issue. Compared with pure Sparing, biodiversity gains are generally more modest under Three-Compartment Sparing (contrast panel (*d*), with (*f*) and (*i*)) because only two-thirds of the area taken out of conventional farming is given completely over to nature restoration, while one-third is converted to low-yield agriculture to support those species reliant on such environments. Again semi-natural grassland endpoints outperform woodland in terms of domestic biodiversity gains, a result echoing findings under the Sharing and Sparing approaches. A further extension of this work would explore the consequences of land-use change for wider ecosystem services. While this is beyond the scope of the present study, previous findings suggest that Sparing strategies tend to outperform Sharing in this respect [71,72].

Our comparison of Sharing and Sparing policies provides a demonstration of the natural capital framework (figure 1) in action. Efficiency concerns (*s*) are considered alongside sustainability analyses (*k*) of the different policy alternatives to be appraised (*l*). Changes to the amount of land conserved, food produced and biodiversity gains were assessed in their natural units (*f*) and cost minimization encapsulated the conversion of agricultural output losses into monetary measures of opportunity costs (*g*). In the absence of the scientific understanding necessary to translate wild species gains from the suite of policy options into monetary units, we quantify them using modelled occupancy predictions and compared aggregate increases under a net gain principle (*i*).

**Figure 4.**
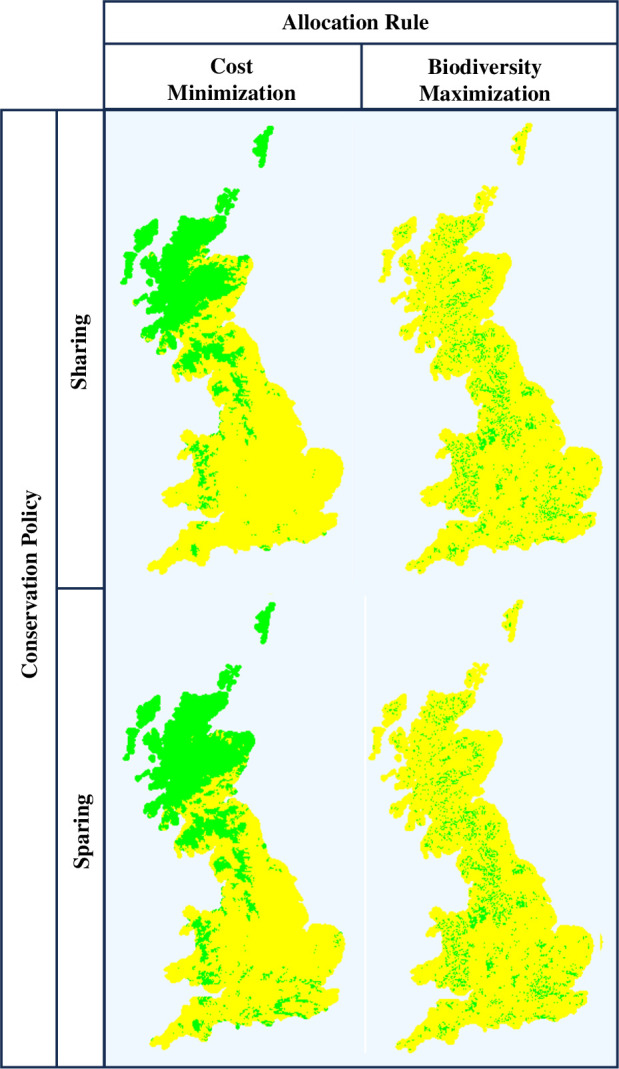
Location of Sparing and Sharing conservation in Great Britain allocated under cost minimization and biodiversity maximization rules. Green pixels represent areas to conserve (via Sparing at 5% Productivity Gain or Sharing with a 600 kha target), while yellow pixels are chosen to remain in production.

A final analysis considers the spatial distribution of changes under different policies and allocation rules. Given that biodiversity is of interest to local populations and that conservation sites can also generate gains in correlated ecosystem services (e.g. access to environmental recreation), spatial distribution provides a basis for equity analyses (*t*). Figure 4 maps the modelled locations of land taken out of production under Sharing or Sparing using either the *Cost Minimization* or *Biodiversity Maximization*. These maps reveal that the Sharing and Sparing policies imply reasonably similar spatial distributions of land removal into conservation within a given approach to implementation, but that conservation locations vary markedly across implementation approaches. *Cost Minimization* focuses conservation on the upland areas of Northern Scotland, Northern England and Central Wales where agricultural output is lowest and farmland is cheapest. By contrast, implementing policies via the *Biodiversity Maximization* rule produces a much more diffuse distribution across all three countries reflecting the variety of areas where species' response to conservation would be greatest.

## 5. Conclusions

The natural capital framework provides a highly flexible and comprehensive approach to bringing the natural environment into conventional economic decision-making. It embraces the diversity of nature’s capital and sets this alongside the manufactured, human and other capital types that dominate policy, showing how the services provided by all combine to generate the goods upon which human well-being rests. The framework also sets out how both private, market-priced and non-market social costs and benefits can be incorporated into analyses and viewed from either an individual/business or public perspective, allowing decision makers to understand the likely private response to changes in public policy and hence refine the latter. Importantly, challenges such as the incorporation of biodiversity are explicitly met through an acknowledgement of the limits to robust economic valuation and incorporation via net gain rules. Application of the natural capital framework provides the necessary information for decision appraisal, taking into account complexities such as spatial variation, temporal change and feedbacks, external effects and the implications of alternative implementation of decisions. Alongside its incorporation of the natural environment within decision support, the natural capital framework provides a rigorous system for bringing the principles of sustainability, efficiency and equity within decision-making.

Here we have used the natural capital framework to contrast different agri-environmental policy approaches to stimulating biodiversity conservation. We examine the currently dominant Sharing approach, which directly removes land from agriculture into conservation, typically in the form of relatively small, unconnected areas. While this enhances domestic biodiversity it does so at the cost of reducing food production, increasing reliance upon imports and raising the prospect of international biodiversity loss. This is compared with a Sparing policy, which stimulates improvements in food yield and uses this to remove relatively larger, connected areas of land into conservation^4^ while keeping overall food production constant and hence avoiding increased reliance upon imports and consequent international biodiversity leakage. A final comparison is made with Three-Compartment Sparing, which again stimulates yield increases to take land into conservation without reducing overall food production. However, unlike the previous Sparing approach, that conserved land is divided between natural habitat and areas farmed at low yield to support specialist species that favour such environments. Under each of these policy types, land is selected into conservation according to three contrasting rules: *Equal Allocation, Cost Minimization* and *Biodiversity Maximization*. Finally, two conservation land-use types are considered: semi-natural grassland and woodland. We apply these analyses at high resolution across the whole of Great Britain, an area comprising significant environmental and economic variation.

Our results suggest, unsurprisingly, that each of these permutations of policy type, implementation method and final land-use matters in that they all have significant effects upon the three overarching decision criteria emphasized through the natural capital framework: sustainability (the degree of biodiversity change delivered); efficiency (the extent of such changes relative to budgets available); and equity (the spatial distribution of changes). Considering impacts on domestic biodiversity, our analyses reveal that, when compared within implementation mode (and in particular when compared across their most suitable implementation mode), even modest improvements in yield under Sparing policies deliver significantly greater biodiversity gains than those provided under extreme levels of area-based conservation through Sharing policies. These results become even stronger when we consider the international biodiversity leakage losses associated with Sharing. The Three Compartment Sparing approach also performs well, again avoiding biodiversity leakage and supporting those specialist species that favour low-yield farmland.

While these results appear unequivocal, the natural capital framework requires that all the major impacts of a proposed policy change should be assessed prior to a decision being made. Nevertheless, assessments to date also favour Sparing approaches (including the Three Compartment Sparing model) over Sharing in terms of the wider ecosystem services and net benefits they generate [51]. The benefits from Sparing and Three-Compartment Sparing stem from increases in yields that mitigate pressures on further land degradation and leakage. Three Compartment Sparing notably delivers food gains and reserves some land in low-yield agriculture aimed at conserving those species that favour such environments (e.g. disturbance-adapted species) rather than purely food production. Given these results and the evidence supporting Sparing and Three Compartment Sparing over Sharing in the literature, perhaps a question for further research concerns why the latter approach has proven so resilient within a policy realm that claims to be evidence-led. Potential explanations could involve the administrative ease of implementing a Sharing approach; that vested interests continue to promote the status quo; or that the negative consequences of Sharing are offshored, beyond the immediate focus of the policy maker. Yet, given the poor performance of Sharing on a range of measures and the urgent need to address the biodiversity crisis, these explanations seem inadequate justifications of the current failure to act.

## Data Availability

This article has no additional data.

## Footnotes

^1^
Akin to other long-term subsidies for land-use change (such as those for afforestation), contracts and monitoring are likely to be required to ensure that Spared land is not subsequently taken back into agriculture [51].

^2^
Alternative approaches might have considered agricultural management practices aimed at delivering biodiversity. However, there is not robust evidence of what these management practices might deliver in terms of species occurrence or agricultural yields, and so our modelling considers land use rather than land management.

^3^
This includes at-risk species from various taxa: Terrestrial mammals (14), Birds (17), Vascular plants (38), Invertebrates (25, of which 10 are butterflies), Lichens (5) and Herptile (1). Future research might contrast at-risk with more common species.

^4^
Such areas are reminiscent of those provided by rewilding. However, the latter approach omits the emphasis upon maintaining food production and avoiding biodiversity leakage losses which characterizes Sparing. Nevertheless, rewilded areas could provide the larger contiguous areas required as a part of a Sparing strategy.

## Dedication

This paper is dedicated to Georgina Mace, a true visionary, wonderful teacher and great friend.
